# Moderate heating renders 7.8-million-year-old sedimentary organic matter bioavailable

**DOI:** 10.1126/sciadv.adw8638

**Published:** 2025-08-20

**Authors:** Shuchai Gan, Verena B. Heuer, Frauke Schmidt, Lars Wörmer, Faming Wang, Rishi R. Adhikari, Patrick Hatcher, Ann Pearson, Kai-Uwe Hinrichs

**Affiliations:** ^1^ MARUM – Center for Marine Environmental Sciences and Department of Geosciences, University of Bremen. Leobener Straße 8, D-28359 Bremen, Germany.; ^2^ South China Botanical Garden, Chinese Academy of Sciences, 510600 Guangzhou, China.; ^3^ Department of Earth and Planetary Sciences, Harvard University, Cambridge, MA 02138, USA.; ^4^ Department of Chemistry and Biochemistry, Old Dominion University, VA 23529, USA.

## Abstract

Marine sediments are a large reservoir of recalcitrant organic matter and host microbes at subsurface depths exceeding 2.4 kilometers and temperatures up to 120°C, yet the mechanisms supplying bioavailable substrates remain unclear. Here, we investigated 7.8-million-year-old sediment from IODP Site C0012 off the Nankai Trough, Japan, through incubations at 20°, 35°, 55°, and 85°C to simulate burial temperatures. Using 3D fluorescence spectroscopy and ultrahigh-resolution mass spectrometry, we tracked changes in dissolved organic matter (DOM). At 35°C, humic-like DOM was released alongside metal ions, exhibiting low bioavailability. At 55°C, abiotic decomposition of humic compounds generated smaller, more bioavailable DOM, promoting fermentation. At 85°C, large nitrogen-containing humic compounds decomposed, producing labile H_2_ and acetate mainly through abiotic processes, bypassing fermentation. Our findings show how abiotic thermal processes activate the refractory organic matter pool, advancing our understanding of long-term carbon sequestration in marine sediments and its implications for global carbon cycling.

## Teaser

Comprehensive analyses of dissolved organic matter illuminate processes that unlock buried carbon in deep Earth.

## INTRODUCTION

Marine sediments are the largest reservoir of organic carbon on Earth (*1*). The accumulation of organic matter in the ocean floor benefits from the biological and microbial carbon pumps, which promote the formation of refractory substances in seawater (*2*, *3*), and the mineral carbon pump, which further protects refractory and labile organic compounds through association with minerals (*4*) and encapsulation in the solid phase (*5*). During sediment burial, diagenesis forms molecularly uncharacterized geopolymers while labile substrates vanish (*6*). Thus, marine sediments have long been considered a pool of refractory organic matter (*7*). However, the discovery of a deep biosphere in the ocean floor (*8*) has challenged this view.

On a global scale, marine sediments harbor as many prokaryotic cells as the overlying seawater (>10^29^) (*9*, *10*). Microbial life has been found as deep as 2.5 km below the seafloor (bsf) (*11*–*13*) and at in situ temperatures of up to 120°C (*14*). Most of subseafloor cells are heterotrophs (*10*, *15*). These extremely slow-growing organisms can use a diverse range of substrates (*15*) and maintain their potential for metabolic activity involved in degrading organic matter in sediments as old as 100 million years (Myr) (*16*–*19*). The realization of the slow but ongoing degradation of refractory organic matter during sediment burial challenges our understanding of the global carbon cycle and has given rise to refined models for the quantification of global carbon fluxes (*18*, *20*), with >90% of the mineralization in subseafloor environments occurring under anoxic conditions (*19*).

In shallow anoxic sediments, biodegradation of organic matter is initiated by extracellular enzymes that hydrolyze macromolecules, so that they can be fermented and mineralized by sulfate reducers, methanogens, and other benthic microbes (*21*, *22*). In deeply buried sediments, organic matter survived the early stages of diagenesis. The sediment matrix also favors its further preservation (*23*). For example, porosity, and thus habitat availability, decreases with depth (*24*, *25*), and mineral-encapsulated organic matter resists extracellular enzymes due to steric hindrance (*26*) and interactions with minerals (*27*). Consequently, the generation of microbial substrates from refractory organic matter in deep, old sediments requires other, still largely unexplored mechanisms.

While the rate at which organic matter is degraded by microorganisms decreases with depth (*28*), temperature increases and exceeds 60°C in nearly 35% of the global marine sediment volume (*29*). Microorganisms survive at temperatures up to 122°C (*14*, *30*–*32*), but little is known about the ecology of mesophiles (20° to 45°C), thermophiles (50° to 80°C), and hyperthermophiles (>80°C) in the nutrient- and energy-limited environment of deeply buried sediments. Above 50°C, thermal cracking of organic matter starts, and, even if sedimentary organic matter contents are low, production of hydrogen and low-molecular-weight organics can provide substrates for microbial life (*11*, *33*–*37*). In the Nankai Trough off Japan, for example, acetate concentrations rise about 450-fold from around 26 μM to 11.6 mM in the porewaters of 3- to 11-Myr-old sediments, as in situ temperature increases from 60°C to >75°C at ~600-m depth (*14*, *34*). The intense, presumably thermal, in situ production of acetate supports a small but highly active population of hyperthermophiles above the sediment-basement interface, which have biomass turnover times of hours to weeks that are extraordinarily rapid for the deep subseafloor biosphere (*31*).

This study aims to better understand how refractory organic matter is transformed into substrates for microbial metabolism during sediment burial and heating. Such transformations are evident in changes of the composition of dissolved organic matter (DOM), but sample requirements for comprehensive DOM analysis exceed the typically available volumes of porewater from deeply buried, compacted sediments. Experiments with sediment samples that are slurried with artificial seawater are an alternative approach (*33*, *38*, *39*), although they cannot perfectly replicate natural biogeochemical conditions. The most obvious deviations are pressure relief, loss of sediment structure during homogenization, and short observation periods. Nevertheless, experiments with sediment slurries allow us to study specific processes and their dependence on individual environmental factors, such as temperature.

Here, we investigated a sample of 7.8-Myr-old sediment retrieved from a depth of 160 m below seafloor (mbsf) in the Shikoku Basin, off the Nankai Trough (fig. S1). At this depth, the redox regime is sulfate reducing. Lithology, organic matter content, and age of the sample resembled the acetate-rich sediments in the Nankai Trough. The in situ temperature, however, did not exceed 24°C, and porewater acetate concentrations were low (~5 μM) (fig. S2). In a 55-day laboratory experiment, we incubated the sediment in slurries with artificial seawater at four temperatures (20°C as the control, 35°, 55°, and 85°C) to simulate three thermal regimes during future burial with conditions favorable for mesophilic, thermophilic, and hyperthermophilic microorganisms, respectively (table S1). We investigated changes in DOM quality as a function of microbial activity and temperature using three-dimensional (3D) fluorescence spectroscopy [excitation-emission matrices (EEMs)] and Fourier transform ion cyclotron resonance mass spectrometry (FT-ICR-MS) and analyzed dissolved manganese, iron, and sulfur to detect evidence for metal and sulfate reduction.

## RESULTS

### Impact of moderate heating on microbial activity, manganese oxide stability, and DOM availability

**Fig. 1.**
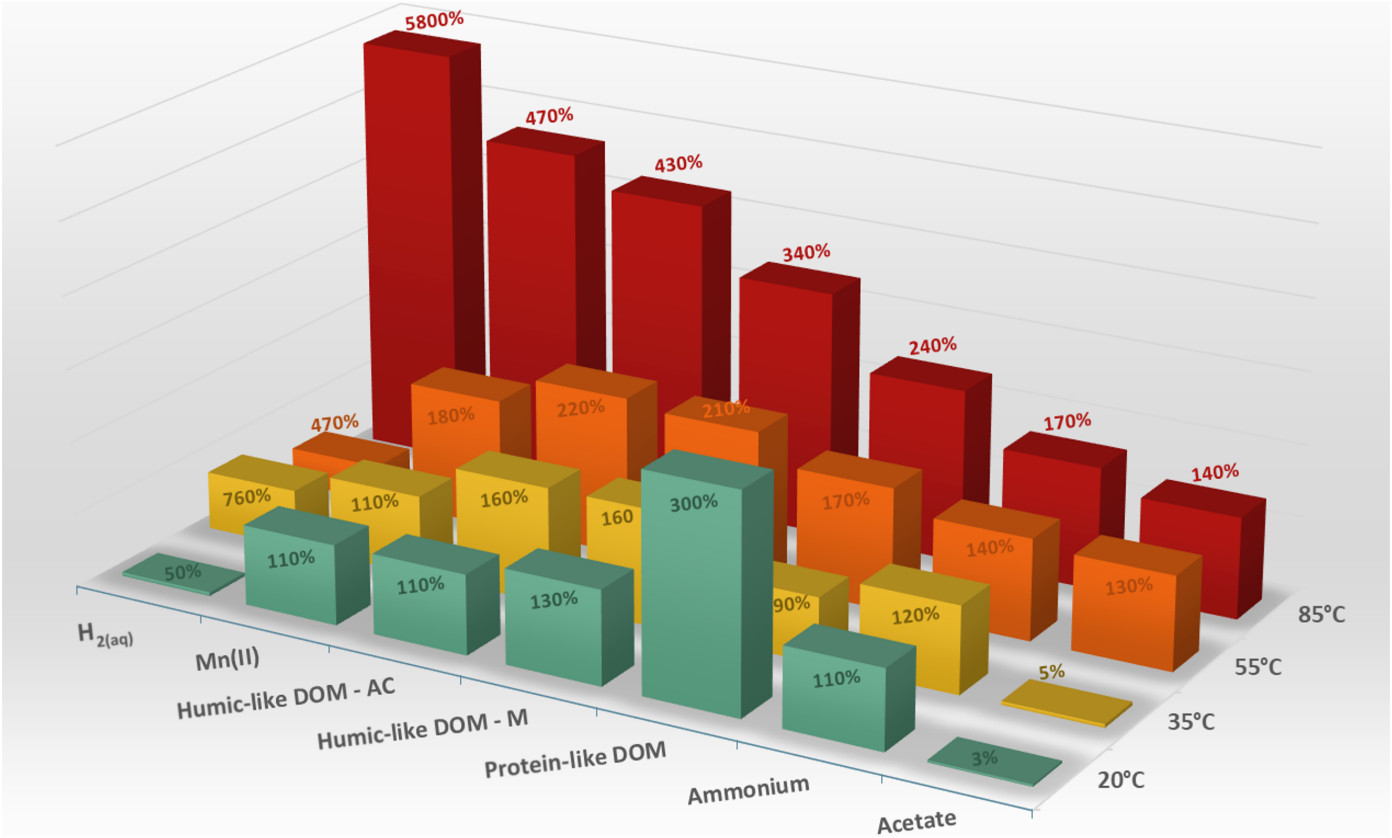
Changes in geochemical parameters after 55-day incubation of 7.8-million-year-old sediment at different temperatures. To facilitate comparison of the various parameters analyzed after 55 days of incubation, data have been normalized to the starting point, i.e., 1 day of incubation at in situ temperature (20°C), with 100% indicating no change, and values of >100 and <100% reflecting relative increase and decrease, respectively. H_2_ concentration changes are displayed on a 1:10 scale to accommodate its larger range in the context of all other analytes. The three dissolved organic matter (DOM) components are identified by EEM spectroscopy and attributed to humic-like DOM peaks AC and M (Hum) and protein-like DOM peak (P). Original (nonnormalized) data are in table S2.

As a proxy for overall biological activity, we determined molecular hydrogen (H_2_) oxidation rates using a radiotracer-based hydrogenase enzyme assay (*40*, *41*) and found a marked >75% decrease at all temperatures ≥35°C compared to the activity at 20°C (fig. S3). The hydrogenase assay shows H_2_ turnover (>10^−7^ mol g^−1^ day^−1^) and thus microbial activity at 20° to 85°C, as well as a pronounced decrease in potential H_2_ oxidation rates with temperature (fig. S3). In close correspondence, dissolved H_2_ concentrations [H_2(aq)_] increased up to 58-fold with temperature (Fig. 1 and table S2).

Concentrations of dissolved manganese [Mn(II)_aq_], humic-like DOM, and ammonium also increased with temperature (Fig. 1), indicating destabilization of manganese oxides and mobilization of organic matter and ammonium from the solid phase of the sediment. After 55 days of incubation, Mn(II)_aq_ concentrations had changed only slightly (~15%) at 20°C but had increased up to 4.7-fold at 85°C (Fig. 1). In contrast, no dissolution of iron (hydr)oxides was observed (fig. S4).

Humic-like DOM was determined together with protein-like DOM by EEM spectroscopy, i.e., 3D fluorescence spectroscopy coupled with parallel factor analysis (PARAFAC). Five individual DOM components were identified on the basis of their excitation (ex) and emission (em) wavelengths (λ). They encompass two humic-like peaks at long wavelengths, with a joint peak AC [λ_ex_/λ_em_: 360(250) nm/460 nm] (fig. S5A) and the individual peak M [λ_ex_/λ_em_: 325(250) nm/400 nm] (fig. S5B), as well as protein-like DOM components, reported together as peak P [λ_ex_/λ_em_: 275 nm/310 nm; 275 nm/350 nm; 250(280) nm/350 nm] (fig. S5, C to E). The humic-like DOM corresponds to the fluorescent properties of humic substances, which are refractory and irregular geopolymers in dynamic association with biomolecular fragments (*42*–*44*). While the humic-like peaks AC and M form a useful proxy for conjugated, soluble geopolymers, with fluorescence overlapping that of fulvic and humic acids (*45*), they do not strictly meet the operational definition of humic acids (soluble in alkaline/neutral but not acidic pH) or fulvic acids (soluble at all pH levels). Peak A is thought to result from aromatic fulvic acids, which are common in natural aquatic systems (*46*). Peak C is more strongly associated with humic acid–like substances and results from large molecules with aromatic functional groups and conjugated systems typically found in terrestrial DOM (*47*–*49*). Peak M is associated with less aromatic and conjugated compounds than peak A and peak C and is commonly referred to as autochthonous, microbial, or marine organic matter with relatively low molecular weights and less conjugation (*47*–*49*).

All humic-like DOM peaks showed an exponential increase that was highly correlated with temperature [coefficient of determination (*R*^2^) > 0.99]. At ≥35°C, the final accumulation of aromatic compounds in peak AC exceeded the increase of smaller and less aromatic compounds in peak M, resulting in an increased AC/M ratio. This shift toward more conjugated compounds led to an overall red shift in the emission spectrum. In sharp contrast to humic-like DOM, protein-like DOM accumulated most strongly at 20°C, where it increased threefold within 55 days of incubation (Fig. 1). At 35°C, it showed a 10% decrease. At 55° to 85°C, however, its 70 to 140% increase paralleled the increase in humic-like DOM (Fig. 1). Overall, the changes in protein-like DOM during 55 days of incubation lack a clear correlation with temperature, suggesting different processing dominates at in situ temperatures and under moderate heating.

The bioavailability of DOM is ultimately reflected in the abundance of low-molecular-weight compounds such as acetate, which serve as substrates for microbes mediating terminal mineralization. Acetate, typically found in concentrations ranging from 1 to 100 μM in marine sediments (*50*), is a key compound in the carbon cycle of anoxic sediments: It is produced by fermentation of organic matter as well as by reduction of CO_2_ via the acetyl–coenzyme A pathway (acetogenesis) and serves as an important substrate for a variety of microorganisms, including sulfate reducing bacteria and methanogens (*50*). When our sediment sample was retrieved, low concentrations of porewater acetate (~5 μM) indicated a balance of production and consumption by fermenters and sulfate reducers, respectively (fig. S2). This balance was disturbed during the preincubation of sediment slurries in rigorously anoxic conditions with 2% H_2_ at 20°C. After preincubation, the slurries were incubated under pressurized N_2_ with an initial acetate concentration of 450 μM. At 20°C and 35°C, the acetate concentration decreased to <20 μM within 55 days of incubation (Fig. 1), thus reestablishing the initial balance of production and consumption. By contrast, acetate concentrations further increased by 30 and 40% at 55° and 85°C, respectively (Fig. 1).

In summary, moderate heating during incubation was associated with reduced stability of manganese oxides and enhanced accessibility of 7.8-Myr-old sedimentary organic matter.

### Temporal evolution of bulk DOM and biological activity

To further elucidate how quantitative and qualitative aspects of DOM evolved during heating, we took samples throughout the 55-day incubation experiment. Additional time series with killed controls, metabolic inhibition of sulfate reducers after 14 days of incubation, and monitoring of H_2(aq)_ and acetate concentrations provide insights into the role of biotic and abiotic processes at each temperature.

**Fig. 2.**
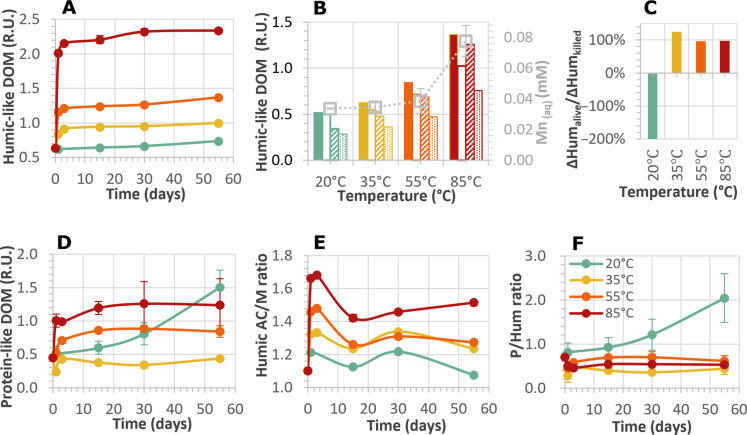
Temporal evolution of temperature-dependent changes in DOM quality and quantity, characterized by EEM spectroscopy, and associated concentrations of dissolved manganese. (**A**) Quantitative changes in humic-like DOM in the live series during 55 days of incubation. (**B**) Corelease of manganese (squares) and humic-like DOM (columns) within 1 day of incubation: AC (filled column) and M (unfilled column) for the killed controls; AC (slashed column) and M (dotted column) for the live series. The exponential regressions with temperature show strong correlations [*R*^2^ > 0.99 for peaks AC and M; *R*^2^ = 0.90 for Mn(II)aq]. (**C**) Increase in humic-like DOM (∆Hum) in the live series normalized to that in killed controls within 1 day. (**D**) Quantitative changes in protein-like DOM in the live series during incubation. (**E**) Changes in humic-like AC to humic-like M ratio (AC/M) and (**F**) Changes in protein-like to humic-like peak ratio (P/Hum) in the live series during incubation. Stage I, 1 day. Stage II, 3 to 15 days. Stage III, 15 to 55 days. Error bars indicate variability in experimental replicates and, in some panels, are smaller than the symbols. R.U., Raman units.

Quantitatively, the time series reveal that the exponential increase in both humic-like DOM components with temperature occurred mainly during the first day of incubation (Fig. 2A and fig. S6), similar to Mn(II)_aq_ (Fig. 2B). At 20°C, humic-like DOM remained relatively stable (Fig. 2A). Accumulation of protein-like DOM (Fig. 2D) proceeded steadily throughout the 55-day long live incubation, which is indicative of biotic production by enzymatic hydrolysis. At 35° to 85°C, the increase in humic-like DOM (∆Hum) within 1 day is similar to that in the killed controls, yielding a ∆Hum_alive_/∆Hum_killed_ ratio of 96 to 125% (Fig. 2C). These findings point to an abiotic process, such as thermal weakening of organic matter–mineral interactions or the evolution of a previously unrecognized abiotic degradation such as mineral catalyzed thermal degradation of the organic matter. At 55° to 85°C, however, there are no signs of an enzymatically driven accumulation of protein-like DOM. Instead, the simultaneous release of protein-like (Fig. 2D) and humic-like DOM within 1 day is in line with a corelease of protein-like DOM that was associated with humic substances (*5*, *51*). This finding suggests that, after several Myr of diagenesis, temperature-driven abiotic processes are spontaneously accelerating the release of refractory and molecularly uncharacterized geopolymers (*6*). Humic substances, namely, geopolymers, are stabilized by hydrogen bonds or through attachment to the solid phase (*52*, *53*), with labile monomers encapsulated in the solid phase (*5*, *51*). A plausible scenario for the underlying processes is the thermal rupture of weak bonds, e.g., ionic bonds and hydrogen bonds, facilitating the spontaneous release of metal ions, humic substances, and ammonia, thus facilitating the accessibility of the encapsulated sedimentary organic matter.

Qualitatively, throughout the 55-day incubation, there were distinct changes in the composition of fluorescent DOM, best seen in ratios of humic-like peaks (AC/M) and P/Hum ratios (Fig. 2, E and F), where P is the fluorescence intensity of the two protein-like components in peak P, and Hum summarizes the fluorescence intensities of humic-like peaks A, C, and M. The proportions of the different humic-like DOM components remained largely unchanged at 20°C but evolved distinctly over time at higher temperatures. We identified three stages of DOM evolution upon heating to ≥35°C. (i) In stage 1, release dominates. Within 1 day, humic-like DOM increased exponentially and a relative increase in the AC peak led to a red shift of fluorescence (Fig. 2E), suggesting that the released humic substances consisted primarily of large molecules with a high degree of condensation. (ii) After 3 days, the release of humic-like DOM ceased, and its subsequent degradation marked stage 2. During this stage (days 3 to 15), the initial red shift was followed by a blue shift, mostly at temperatures ≥55°C, indicating the loss of conjugated structures such as aromatic rings, covalent bonds, and adjacent functional groups acting as auxochromes. Killed controls showed the consistent evolution of AC/M ratios over time and with temperature (fig. S7), thus confirming the abiotic nature of the underlying processes. At 55° and 85°C, P/H ratios increased again during the subsequent blue shift of humic-like DOM (Fig. 2F), suggesting a formation of protein-like DOM during the decomposition of humic-like DOM in stage 2. (iii) Toward the end of the experiment, the formation of refractory DOM marked stage 3 (days 15 to 55) at 85°C, in which the formation of more aromatic and conjugated compounds led to a second red shift (Fig. 2E).

**Fig. 3.**
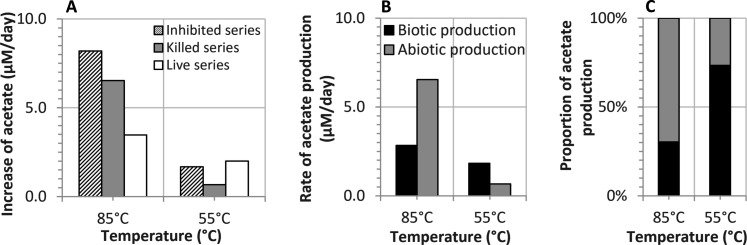
Production of acetate during incubation at 55° and 85°C. (**A**) Rate of acetate accumulation in live, killed, and inhibited series (0 to 15 days for the killed series; 0 to 55 days for the live and inhibited series). (**B**) Rates of biotic and abiotic acetate production calculated from the live, killed, and inhibited series. (**C**) Relative contribution of biotic (black column) and abiotic (gray column) acetate production [same legend as in (B)].

Consistent with hydrogenase activity, the time series of H_2(aq)_ and acetate confirm metabolic activity in all live incubations. Although hydrogenases were markedly reduced above 35°C, H_2(aq)_ remained <10 nM and thus within the thermodynamic threshold concentration of H_2_ consumers (*54*) throughout all live incubations (fig. S8A). Inhibition of sulfate reducers, accompanied by decreased hydrogenase activity (fig. S3), led to a transient accumulation of H_2(aq)_ at 20° to 35°C and a steady increase of H_2(aq)_ at 85°C (fig. S8B). In the killed controls, H_2(aq)_ remained low at 20° to 35°C but was 15-fold higher than that in the live incubation at 85°C (fig. S8C). These findings indicate not only considerable abiotic production of H_2_ at 85°C but also its rapid and nearly balanced microbial consumption. Likewise, at 85°C, abiotic acetate production was rapid (Fig. 3) and exceeded microbial consumption, leading to increase in acetate concentrations. Notably, the inhibition of sulfate reducers and the killed control at 55°C did not lead to as rapid an increase in H_2(aq)_ (fig. S8) or acetate (Fig. 3 and fig. S9) as observed at 85°C. This indicates that the production of intermediates at 55°C, either by fermentation or abiotic processes, was slower than at 85°C.

### Changes in molecular composition of DOM

**Fig. 4.**
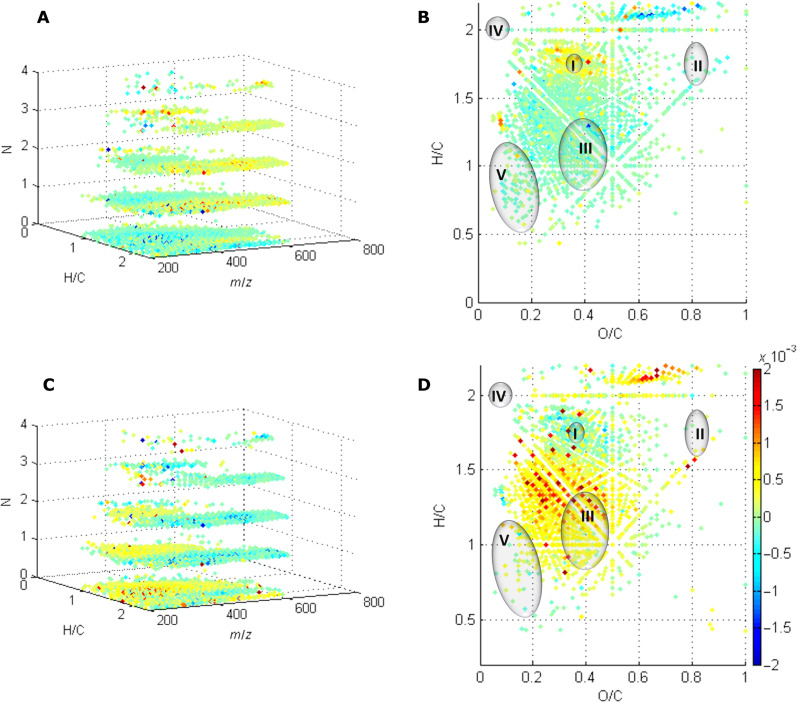
Changes in the molecular composition of DOM, characterized by FT-ICR MS, during 55 days of incubation at 85°C. (**A**) Formula changes after 1 day of incubation, where the *z* axis shows the number of nitrogen atom and *m*/*z* shows the mass distribution; (**B**) Van Krevelen diagram of formula changes after 1 day of incubation; (**C**) formula changes from day 1 to day 55, where the *z* axis shows the number of nitrogen atom and *m*/*z* shows the mass distribution; (**D**) Van Krevelen diagram of formula changes from day 1 to day 55. Dot colors in all panels [(A) to (D)] indicate changes in relative intensity (rInt), scaled with a shared color bar: Positive values and negative values represent increased and decreased normalized intensity of formulae, respectively. The circles in the Van Krevelen diagram represent the potential range of biomolecular components, protein (I), cellulose (II), lignin (III), lipids (IV), and condensed aromatics (V) (*85*, *86*). Note that geopolymers produced during diagenesis of biomolecules are not necessarily located in the corresponding regions.

To better understand the temperature-driven changes in DOM quality, we determined the molecular composition of the initial DOM pool (20°C, start) by FT-ICR-MS and investigated changes in molecular formulae after 55 days of incubation at 35° and 55°C, as well as changes after 1 and 55 days of incubation at 85°C (Table 1). In the initial DOM pool before incubation, 5030 formulae were assigned. The molecular composition of DOM was high in hydrogen-bearing compounds and low in oxygen- and nitrogen-bearing compounds. H/C_wa_ and O/C_wa_ ratios were 1.5 and 0.38, respectively, and 40% of the formulae contained nitrogen, mainly CHNO-N1 and CHNO-N2, yielding a low weighted-average nitrogen-per-molecule content (N_wa_) of 0.5 (Table 1). After 55 days of incubation at 35° and 55°C, the average size of the DOM molecules had slightly increased without pronounced changes in the average molecular formula composition (Table 1). At 85°C, however, larger DOM molecules were already released after 1 day of incubation, increasing the weighted-average carbon content (C_wa_) distinctly from 19.0 to 22.3 and the weighted-average mass-to-charge ratio (*m*/*z*_wa_) from 383 to 444 (Table 1). The freshly released DOM was more saturated and less oxygenated (Table 1). Both aliphatic and aromatic compounds were released (Fig. 4). Accumulation of nitrogen-bearing compounds (CHNO) at higher molecular weights (Fig. 4A) increased the N_wa_ (Table 1), and the most increased formulae fell into the protein-like region of the Van Krevelen diagram according to their H/C_wa_ and O/C_wa_ ratios (Fig. 4B). CHNO-N1 and CHNO-N2 compounds dominated and were accompanied by a few CHNO-N3 and CHNO-N4 compounds (Fig. 4A). CHNO-N1 and CHNO-N2 molecules can be formed from semidegraded peptides (*55*) via deamination (*56*) or the incorporation of N into CHO fraction (*57*, *58*). During further incubation at 85°C, the number of CHNO-N3 and CHNO-N4 formulae declined and the molecular weight of DOM corresponding to the CHNO-N1 and CHNO-N2 formulae decreased, while N-depleted compounds accumulated in the lower molecular weight range (Fig. 4C). The release and breakdown of large aliphatic CHNO-N1 and CHNO-N2 compounds are consistent with the observed changes in AC/M ratios (Fig. 2) and validate their interpretation. In addition, double bonds and aromatization increased, and the associated loss of H atoms (Table 1) is consistent with the large increase of abiotic H_2_ production at 85°C (fig. S8). It is intriguing to explain the decomposition of CHNO compounds. Although humic substances are considered complex molecules, they are composed of mainly C–H, C–C, C=C, C–O, C=O, and C–N bonds (*42*, *52*, *59*). The breakdown of large aliphatic CHNO-N1 and CHNO-N2 into smaller molecules may be attributed to the cleavage of the C–N bond. C–N bonds are more prone to breaking than C–O and C–C bonds, as bond energies increase in that order (*60*).

**Table 1.** Intensity-weighted averages (“wa” in subscript) of characteristic parameters derived from FT-ICR-MS analysis. Formulae with ^13^C or ^34^S were not included. Intensity-weighted average values (*X*_aw_) were calculated according to equation: *X*_aw_ = ∑*X*_i_ × rInt_i_/∑rInt_i_.

Sample	*m*/*z*_wa_	C_wa_	H_wa_	O_wa_	N_wa_	S_wa_	P_wa_	DBE-O_wa_	H/C_wa_	O/C_wa_
20°C, start	383.3	18.9	28.0	6.79	0.50	0.36	0.05	−0.57	1.51	0.38
35°C, end	404.3	20.2	28.7	7.36	0.54	0.26	0.03	−0.23	1.44	0.38
55°C, end	397.4	19.9	28.3	7.03	0.57	0.28	0.06	0.02	1.44	0.37
85°C, 1 day	444.3	22.3	37.5	6.96	1.21	0.30	0.06	−1.75	1.68	0.33
85°C, end	413.1	20.8	31.2	6.92	0.89	0.27	0.06	−0.26	1.50	0.35

### Implications for mechanisms of organic matter degradation in deeply buried sediments

Our study shows how temperature affects both the release of DOM from 7.8-Myr-old sediment and its subsequent transformation, with important implications for microbial life and carbon cycling in deeply buried sediments. At 20°C, a steady biotic production of protein-like compounds determined the composition of fluorescent DOM, while the release and degradation of humic-like DOM was negligible. Biological activity was high enough to facilitate rapid turnover of H_2_ and acetate. Thermodynamically controlled acetate concentrations of ~11 μM are consistent with the observed dominance of sulfate reducers and closely resemble in situ acetate concentrations in the interstitial waters of the sediment. These findings suggest that release and degradation of DOM are ongoing in the 7.8-Myr-old sediment.

At 35°C, the degradation of protein-like DOM outweighed production. Humic-like DOM was released upon heating and showed no signs of transformation during the course of the experiment. Nearly constant AC/M ratios and the invariable molecular composition suggest a rather recalcitrant nature of DOM at this temperature. Biological activity was reduced by 75% compared to the in situ temperature of 20°C but was still sufficient to balance the biotic production of H_2_ and acetate by fermenters, which rely on the degradation of organic matter through extracellular enzymes and on consumption of their degradation products by sulfate reducers and other terminal oxidizers.

At 55°C, heating resulted not only in a notable corelease of protein-like and humic-like DOM but also in a subsequent decomposition of humic-like DOM that went along with an increase in protein-like DOM and with the accumulation of acetate. In spite of these quantitative changes, there was no noticeable change in the molecular DOM composition. The limited acetate production in the killed control points to biotic acetate formation (Fig. 3), and H_2_ concentrations too low (<5 nM) to make acetogenesis thermodynamically feasible (*50*) suggest acetate production via fermentation of larger precursor molecules. Fermentation, however, requires cell-accessible precursors. Previous studies have shown that glucosidase and protease enable biological hydrolysis and support acetate production at ~55°C (*61*), but the enzymes denature rapidly at this temperature (*62*) with a short half-life of ~17 to 45 hours (*63*). To test whether hydrolysis is a limiting factor for fermentation at 55°C, we conducted additional experiments in which the sediment slurry was amended with either yeast extract (YE) as an additional source of protein-like DOM or with prehydrolyzed YE. At 35°C, both series showed similar consumption of protein-like DOM during 10 days of incubation (fig. S10). At 55°C, however, the degradation of protein-like DOM increased by 40% when the YE was artificially hydrolyzed before incubation (fig. S10), thus underlining that hydrolysis may be the rate-limiting step in the degradation of biopolymers at 55°C. We suggest that the thermal breakdown of humic-like DOM at 55°C may provide suitable substrates for fermenters, thereby compensating for the decline of biotic hydrolysis at this temperature.

At 85°C, the spontaneous corelease of manganese, protein- and humic-like DOM further increased, and thermal processes changed the molecular composition of DOM markedly throughout the experiment. The freshly released DOM consisted of high-molecular-weight compounds with 1 to 4 nitrogen atoms and a high degree of saturation. Their thermal breakdown formed nitrogen-depleted molecules in the lower-molecular-weight range. In addition, the number of double bonds and aromatization increased. Simultaneously, abiotic formation of H_2_ and acetate led to an accumulation of both compounds.

## DISCUSSION

Our experimental results compare well to previous findings in up to 120°C hot and 1.2-km-deep sediments in the Nankai Trough. At IODP Site C0023, concentrations of vegetative cells decrease by two orders of magnitude above 45°C, acetate concentrations increase ~450-fold as the in situ temperature rises from 60° to >75°C, and a tiny but highly active population of hyperthermophiles persists at 100° to 120°C above the sediment-basement interface (*14*, *31*). The rapid decline in the H_2_ oxidation potential and, consequently, the overall biological activity with increasing temperature, as observed in the incubation experiment, are analogous to the in situ decline in vegetative cell concentrations with depth at Site C0023. This observation lends support to the hypothesis that temperature is the primary factor controlling organic matter reactivity rather than sediment age in the deep seafloor. On the basis of our experimental results, we argue that temperature-dependent changes in the bioavailability of the sedimentary organic matter may be involved. The 7.8-Myr-old sedimentary organic matter is still biodegradable at temperatures ~20° to 35°C, but, at 55°C, the biotic hydrolysis of macromolecules by extracellular enzymes decreases, from either a decline in enzyme stability or a reduction in the production of extracellular enzymes. In either case, this results in a diminished supply of monomers that can be used by fermenters. Thus, this temperature window is particularly unfavorable for microbial processes, including fermentation and terminal mineralization. At temperatures above 55°C, abiotic processes enhance the bioavailability of DOM and notably contribute to the production of H_2_ and acetate, which can both serve as substrates for sulfate reducers and methanogens. The total released acetate, which represents only a fraction of bioavailable carbon, was estimated to correspond to ~0.05% of total organic carbon at 55°C and ~0.25% at 85°C. Inhibited series demonstrated that acetate accumulation rates increased markedly with temperature, rising from 1.7 μM/day at 55°C to 8.2 μM/day at 85°C (Fig. 3). In situ measurements at IODP Site C0023 revealed an acetate concentration of 9.2 ± 2.4 mM (*14*). Based on the experimentally determined production and consumption rates and assuming consistent acetate production without additional sinks, we estimate that it would take ~10 years for acetate to reach its observed in situ concentration at temperatures of 85°C or higher.

Our results support the view that the reactivity of sedimentary organic matter is determined not only by its molecular composition but also by environmental factors (*35*), with heating during sediment burial leading to the production of hydrogen and acetate serving as substrates for microbial life in the deep biosphere (*33*, *34*). Heating has been shown to induce shifts in the activity and composition of microbial communities involved in terminal metabolic processes (*38*). Our results provide additional insights into the initial and intermediate steps of organic matter degradation and suggest that the biotic degradation chain becomes fragmented at higher temperatures, with abiotic hydrolysis and production of intermediates relaxing the need for symbiotic relationships in the thermophilic microbial community. Consequently, the dependence of terminal mineralizers, e.g., acetate and hydrogen utilizers, on hydrolyzers and fermenters decreases with increasing temperature. In addition, abiotic hydrolysis and production of intermediates could facilitate organic matter degradation in consolidated sediments, where reduced pore size hinders enzyme accessibility.

The conclusions drawn from the incubation of organic-matter lean Shikoku Basin sediments in this study agree well with recent findings in up to 120°C hot and organic-matter rich sediments of the Guaymas Basin, a young marginal rift basin in the Gulf of California. In Guaymas Basin, microbial sulfate reduction rates were high in the temperature range of thermophiles and hyperthermophiles but showed a marked gap at 65° to 75°C (*64*), which coincides with the substrate bottleneck observed in this study. In addition, DNA recovery was markedly low at 50° to 60°C (*65*, *66*), too. Despite the presence of organic-matter rich sediments, the activity of sulfate reducers and the size of the microbial community may be limited by substrate availability due to the temperature sensitivity of the extracellular hydrolases that produce fermentable substrates.

**Fig. 5.**
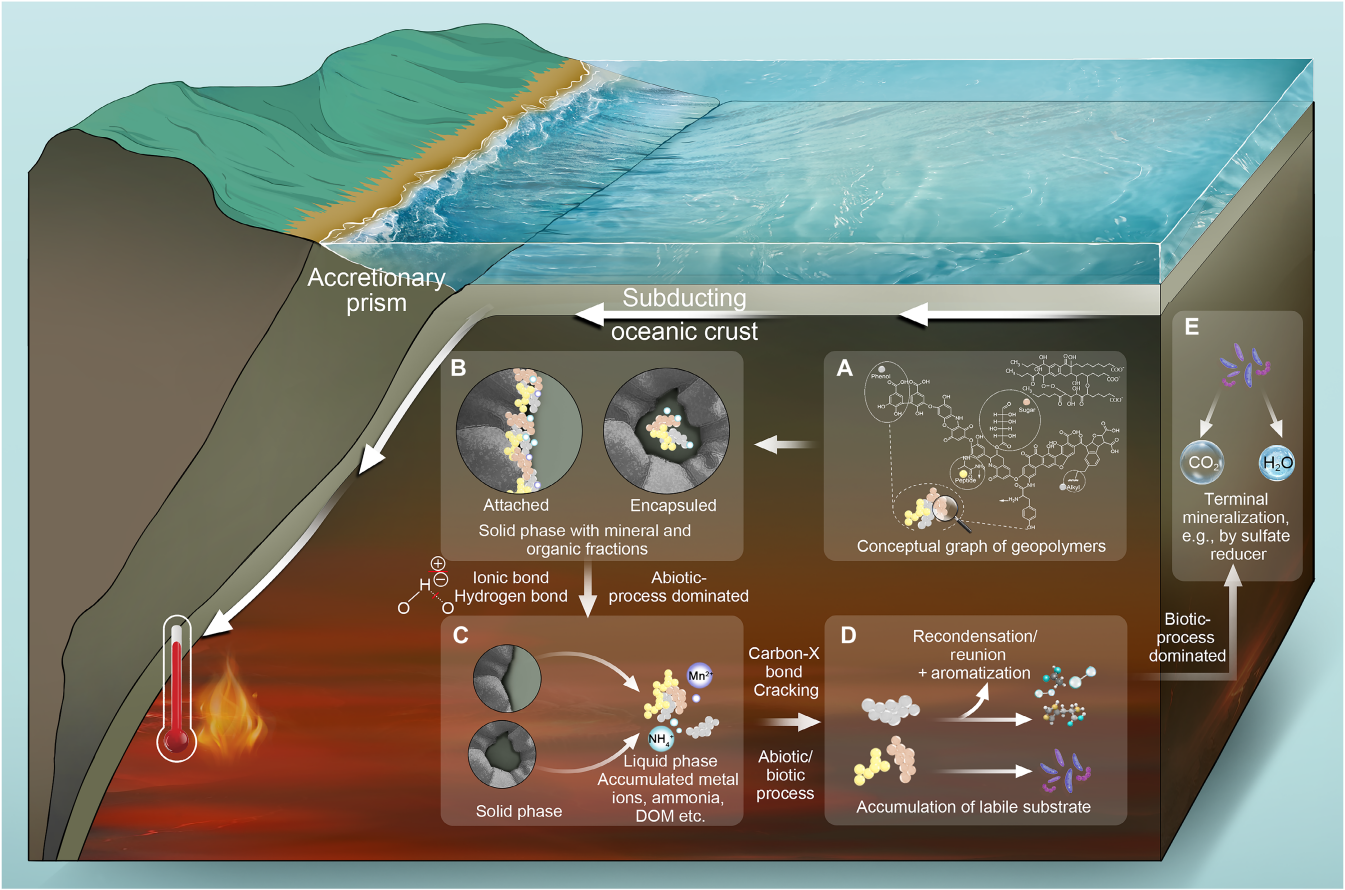
A proposed model for the abiotic decomposition of humic substances due to heating. (**A**) Model of a humic substance (left), adapted from Stevenson’s model (*42*) with insights from Leenheer and Rostad (*43*); model of humic acid in seawater (right), based on the model by Harvey *et al*. (*87*), reprinted with permission from Elsevier; (**B**) humic substances attached to minerals and labile compounds trapped in humic substances in the solid phase; humic substances adsorbed on solid-phase minerals contribute to the formation of hydrogen or ionic bonds between clay minerals and ammonia (*88*); (**C**) humic substances were detached from the solid phase, and labile compounds trapped in humic substances were released; (**D**) large humic substances were decomposed due to heating, and during decomposition, the small monomers were mainly produced abiotically and assimilated by microbes above 55°C; metabolites were mainly produced abiotically and aromatics accumulated by 85°C; (**E**) terminal mineralization by microbes.

The mineral carbon pump is the driving force behind the sequestration of organic matter in soils and surface sediments. It enhances stability and accumulation through a range of organic matter-mineral interactions, including adsorption, occlusion, aggregation, and polymerization (*4*). Our study shows how moderate heating to temperatures ≥35°C can apparently reverse the mineral carbon pump in deeply buried sediments (Fig. 5). Upon heating, the rapid relaxation of organic matter-mineral interactions leads to a spontaneous corelease of Mn(II)_aq_, humic-like DOM, protein-like DOM, and ammonium, and thus improves their availability to microbes. Furthermore, the rapid release of humic-like DOM shows similar trends in both alive and killed controls (Fig. 2B), indicating a controlling role of abiotic processes. Old and recalcitrant organic macromolecules are subsequently transformed by abiotic processes at moderate temperatures. This is suggested by a loss of conjugation structures in both alive and killed series (Fig. 2E and fig. S7), which shows in decreasing AC/M ratios of fluorescent DOM at ≥35°C, by the production of protein-like compounds at ≥55°C and by the formation of acetate and a decreasing molecular weight of DOM formulae at 85°C. The abiotic transformation of refractory organic matter into a more labile DOM pool resembles a reversal of the microbial carbon pump. The microbial carbon pump is a key mechanism for global carbon storage in the ocean, whereby microbes convert labile DOM into refractory organic matter (*2*). In this study, however, thermal breakdown of refractory organic matter converts it abiotically back into more bioavailable DOM, thereby facilitating its microbial utilization in the deep biosphere. Our findings indicate that carbon cycling in deep Earth environments differs from surface processes, notably in resembling a reversal of the mineral carbon pump above 35°C and the microbial carbon pump above 55°C, and both largely accelerated at 85°C accompanied by the formation of a reprocessed refractory carbon pool.

Our experimental investigation of the impact of temperature on DOM in 7.8-Myr-old sediment sheds light on processes that could potentially unlock buried organic carbon deep within the Earth. These processes are consistent with observations of microbial substrates and activities in deep, hot sediments of the Nankai Trough and Guaymas Basin, which were accessed through scientific ocean drilling. Nevertheless, we note that our laboratory study could not fully mimic the complex nature of a natural system that experiences heating during sediment burial under high pressure conditions, with lower heating rates, and within a compact sediment matrix. While the culturability of microbial communities may not be strongly pressure sensitive (*67*, *68*), long-term geochemical and mineralogical processes, such as clay recrystallization (*69*), were not captured on the incubation timescale. Slurry preparation may also loosen sediment structure and alter substrate-mineral interactions (*39*), affecting the extent of DOM accumulation. Nevertheless, isolating temperature as a single variable allowed us to identify temperature-driven mechanisms of organic matter transformation and the abiotic-biotic interplay.

In conclusion, our study shows that moderate heating leads to a bifurcation of the DOM pool. One pool becomes increasingly bioavailable, consisting of smaller, aliphatic CHNO compounds that are readily metabolized by microorganisms. The other pool becomes a more refractory reservoir, characterized by aromatic compounds that contribute to long-term carbon storage. In particular, higher temperatures enhance the production of labile substrates by abiotic processes. While the seafloor has long been recognized as an important reservoir of refractory organic carbon preserved over geological timescales, our results suggest a dynamic interplay between this carbon pool, the deep biosphere, and physical factors such as temperature, highlighting the complex nature of carbon cycling in deep subsurface environments.

## METHODS

### Sampling of Site C0012

Site C0012 (32°44.8880′N, 136°55.0240′E; water depth, 3510.70 m) was established with the drilling vessel DV Chikyu during Expedition 322 of the International Ocean Drilling Program (IODP) in the context of the Nankai Trough Seismogenic Zone Experiment (NanTroSEIZE). The site is located in the Shikoku Basin (fig. S1), the back-arc basin of the IZU-Bonin volcanic chain, and serves to characterize sediments before their subduction in the Nankai Trough. At Site C0012, a 538-m-thick layer of sediments has accumulated on top of the Philippine Sea plate over a period of ~19 Myr, containing early Miocene to Quaternary microfossils (*70*). The sediments were deposited under low sedimentation rates. The upper layers are dominated by marine organic matter, while terrestrial input becomes more pronounced at greater depths (>~300 m) (*70*). The seafloor temperature is ~2.85°C, and the thermal gradient averages 135°C/km (*71*, *72*). The organic carbon content of the mainly hemipelagic sediments is very low (0.31 ± 0.17 wt %), and so is the microbial activity. The sulfate-reduction zone extends down to ~200 mbsf, and, at greater depths down to the basement, concentrations of dissolved methane do not exceed 250 μM (*70*). Functional gene analyses for deeper layers (>~300 m) at Site C0012, under conditions consistent with sulfate reduction, revealed a phylogenetically diverse sulfate-reducing community dominated by *Desulfovibrionales* and *Desulfobacterales*, most of which are distantly related to known cultured strains (*73*).

For the incubation experiment, a 10-cm whole-round-core sample (C0012A-13R-2 WR, 5.0 to 15.0 cm) was collected from a freshly cut core. The sample originates from a layer of hemipelagic mudstone recovered from 159.69 to 159.79 mbsf. The sediment, ~7.8 Myr old, had a bulk density of 1.82 g/cm^3^, a porosity of 56%, an in situ sulfate concentration of 10.45 mM, a predominantly marine source of organic matter (*70*), and a temperature of ~24°C (*72*). The sample was immediately processed in an anaerobic chamber onboard DV Chikyu. To prevent potential contamination from drilling fluid, the outer part of the whole-round core was removed with a sterile knife before the consolidated sediment was crushed and transferred into a sterile 500-ml glass bottle. The bottle was closed with a butyl rubber stopper, flushed with nitrogen to maintain anaerobic conditions, and stored at +4°C in the dark.

### Preparation of sediment slurry

The sediment was mixed with artificial seawater to generate a 7:5 (w:w) slurry. The artificial seawater was prepared with a 15 mM sulfate concentration to match the genuine sulfate concentration of the sample. It was autoclaved, flushed with N_2_, and supplemented with vitamins, trace elements, and bicarbonate to protect and stimulate anaerobic microorganisms. The exact composition of the artificial seawater is detailed below. To regulate redox conditions, thiosulfate and sulfide were added to the slurry to final concentrations of 133 and 500 μM, respectively. Nutrients were supplemented to achieve ~1 mM ammonium and ~0.3 mM phosphate in the artificial seawater.

Due to the advanced consolidation of the sediment, it took a period of 4 weeks to prepare, homogenize, and preincubate the slurry, before it was lastly split into subsamples. The crushed mudstone was first mixed with artificial seawater inside an anaerobic chamber (N_2_ atmosphere with 2% H_2_). The mixture was sealed in a sterile plastic bag, homogenized by continually malaxating the bag for 2 hours, and transferred into a sterile 1-liter Schott bottle. The bottle was closed with a sterile butyl rubber stopper inside the anaerobic chamber to maintain a N_2_ atmosphere with 2% H_2_. The mixture was preincubated at room temperature, which resembles the in situ temperature of ~24°C. After 1 week, the headspace was replaced by pure N_2_, and the preincubation in Schott bottle at room temperature was continued. After another week, sediment mudstone was softened, and the slurry was homogenized again by malaxating in a sterile bag. The slurry was split into 28 subsamples and transferred into eight 250-ml Schott bottles and 14 20-ml Hungate tubes inside an anaerobic chamber, yielding a final ratio of 3:1:1 (v:v:v, headspace:medium:sediment). The bottles and tubes were sealed with sterile butyl rubber stoppers (boiled for 2 hours in 0.1 M NaOH and three times in Milli-Q water) and flushed with N_2_ to keep the slurry under N_2_ at a pressure of 2 bar. The subsamples were incubated at room temperature for another 2 weeks until the main experiment started. All work was conducted under sterile conditions.

### Main incubation experiment

The subsamples were incubated in four temperature series (table S1) at 20°C (control, corresponding to the temperature in situ), 35°C (favorable for mesophiles), 55°C (favorable for thermophiles), and 85°C (favorable for hyperthermophiles). Each temperature series consisted of three subseries, namely, one with untreated sediment (alive), one in which sulfate reducers were inhibited by addition of molybdate (20 mM) after 15 days of incubation (inhibited), and one in which the sediment slurry was autoclaved (120°C) twice before incubation to stop microbial activity (killed). Each subseries comprised two replicates, except for the killed controls at 20° and 85°C, which could not be duplicated due to sample limitation. The experiment started on day 0 with a gradual increase of each incubator temperature by 10°C/hour. Once the target temperatures were reached, the headspace was flushed with N_2_ for 5 min, the pressure was adjusted to 2 bar, 0.5 ml of the headspace gas was taken for H_2_ analysis, and the liquid phase was sampled and processed as described below. Subsequently, H_2_ analyses were conducted twice per week throughout the experiment. Liquid-phase sampling was repeated after 1, 3, 15, 30, and 55 days. Due to sample limitation, however, large-volume samples for characterizing DOM by FT-ICR MS were only collected on days 0 and 55. An exception is the 85°C series, in which an FT-ICR MS sample was also collected after 1 day of incubation. The additional sample facilitates insights into short-term effects at the highest incubation temperature. After 55 days, the experiment was stopped, and the remaining sediment slurries were preserved for determination of hydrogenase activity through storage of the closed incubation vials at −80°C.

### Adjustments during the incubation experiment

On the basis of our observations during the incubation experiment, we modified our experimental plan in two ways: (i) Long-term incubations of killed controls can suffer from the reactivation of dormant cells, such as endospores (*74*, *75*). We observed recovery of microbial activity in our killed controls after 3 weeks of incubation. Consequently, killed control data are only available for samples taken on days 0, 1, 3, and 15, and hydrogenase activity was not determined at the end point. (ii) During the initial phase of the experiment, EEMs revealed changes in protein-like peaks, supposedly due to temperature dependent changes in the hydrolysis of biopolymer fragments. To further elucidate this effect, we implemented two complementary experiments with YE, because YE shows a typical protein-like peak in EEMs. In one subseries, the sediment slurry was supplemented with YE (1 mg/liter); in the other subseries, it was supplemented with YE that had first been hydrolyzed for 16 hours in 85°C hot and oxygen-free 4 M NaOH under pure N_2_. For both subseries, two replicates were prepared and incubated for 10 days at 35°, 55°, and 85°C. The EEMs were sampled at the start and at the end of the experiment.

### Collection of liquid-phase samples

Liquid-phase samples were collected in a glass vial and immediately split and processed in an anaerobic chamber. Subsamples for acetate analysis were filtered using nylon filters (0.2 μM, Omni lab). Subsamples for other analyses were filtered using cellulose acetate filters (0.2 μM, Sartorius AG). All vials were sealed in the glovebox and covered with Parafilm afterward. Samples for EEMs, acetate (in glass vials), and inorganic ion (in plastic tubes) analysis were stored at −20°C. Twenty-milliliter samples were collected for each FT-ICR MS analysis. Before measurement, samples were acidified to pH 2, and DOM was extracted using styrene-divinylbenzene polymer-based cartridges (Agilent, 3 ml, 200 mg) under N_2_ atmosphere to remove salts (*76*). The cartridges were eluted with 1.5 ml of methanol (Lichrosove quality, Merck), and the eluent samples were stored at −20°C in a Schott bottle filled with N_2_. Solid-phase extraction caused selective compound loss, with ~40% DOC recovery. Protein-like DOM was recovered at similar efficiency to total DOC, while humic-like DOM showed higher recovery (*77*, *78*). As a complementary technique to FT-ICR MS, EEMs preserved signals of compounds typically lost during extraction, such as protein-like DOM, and enabled high-resolution analysis using just 50 to 100 μl of porewater (*77*).

### Analysis of hydrogen oxidation potential

The sediment slurry was incubated under tritium gas to determine the potential activity of hydrogenase enzymes, which catalyze isotopic exchange between H_2_ and water molecules. The radioactivity of tritiated water was measured by a liquid scintillation counter (PerkinElmer, Tricarb 2810TR). The method is described in detail elsewhere (*40*, *41*). The data result from a radiotracer-based hydrogenase enzyme assay that builds on the central role of molecular hydrogen (H_2_) in the anaerobic degradation of organic matter. Hydrogenases are ubiquitous enzymes in microorganisms that use H_2_ as a substrate, intermediate, or product. When incubated under tritium (^3^H_2_) gas, they catalyze isotope exchange with water molecules. The production of tritiated water (^3^H_2_O), measured by liquid scintillation counter, is a sensitive measure of potential H_2_ oxidation rates and thus of microbial activity irrespective of the predominant electron accepting process (*40*, *41*).

### Analysis of H_2_ concentration

The partial pressure of H_2_ in the headspace gas of the incubation vials was measured on a Peak Performer 1 gas chromatograph (Peak Laboratories, USA) as described previously (*79*). The concentration of dissolved H_2_ in the liquid phase of the slurry was derived following the approach of Crozier and Yamamoto (*80*). The solubility coefficient β was calculated according to the following equation

$$\begin{array}{c} \text{ln}\left(\beta \right)=A1+A2*\left(100/T\right)+A3*\text{ln}\left(T/100\right)\\ +S*\left[B1+B2*\left(T/100\right)+B3*{\left(T/100\right)}^{2}\right] \end{array}$$

where A1 = −39.9611; A2 = 53.9381; A3 = 16.3135; B1 = −0.036249; B2 = 0.017565; B3 = −0.0023010; and *T* and *S* represent absolute temperature and salinity, respectively.

### Analysis of acetate concentration

The concentration and stable carbon isotopic composition of acetate in the liquid phase of the slurry was determined by isotope ratio monitoring liquid chromatography/mass spectrometry using a Surveyor HPLC (ThermoFinnigan) combined with an LC IsoLink interface (ThermoFinnigan) and a DELTA Plus XP mass spectrometer (ThermoFinnigan) as described previously (*14*, *50*, *81*).

### DOM characterization by EEM

Samples were measured on a fluorescence spectrophotometer (Agilent Cary Eclipse, USA) after dilution with O_2_-free Milli-Q water adjusted to the salinity of the sample (35 g of NaCl per liter). Ninety-three samples were modeled by PARAFAC. Five components were identified with the following wavelengths (λ) of excitation (ex) and emission (em): Two protein-like peaks (λ_ex_/λ_em_: 275 nm/310 nm; 275 nm/350 nm) are reported together as peak P. Two humic-like peaks are reported together as peak H and individually as peak M [λ_ex_/λ_em_: 325(250) nm/400 nm] and peak AC [λ_ex_/λ_em_: 360(250) nm/460 nm]. The wavelength number in the parentheses of the λ_ex_/λ_em_ data refers to the minor peak. One peak was found in the artificial seawater used for slurry preparation [λ_ex_/λ_em_: 250(280)/350 nm]. It is a critical component of the blank, as it overlaps with one of the protein-like peaks in the samples. We attribute this peak to fluorescence emitted by the vitamin solution. In the final dataset, these signals are corrected by blank-subtraction. Peak AC displays joint peaks A and C, both characterized by longer emission wavelengths and higher conjugation than peak M. Fluorescence intensity is reported in Raman units.

### DOM characterization by FT-ICR MS

The eluent was diluted to a DOM concentration of 20 mg of C/L in methanol:water (1:1, v/v). Samples were ionized with electrospray ionization (Apollo II electrospray source, Bruker Daltonik GmbH, Bremen, Germany) and characterized in negative ion mode on a Bruker SolariX XR FT-ICR-MS (Bruker Daltonik GmbH, Bremen, Germany) equipped with a 7-T refrigerated actively shielded superconducting magnet (Bruker Biospin, Wissembourg, France). The mass spectrometer was calibrated with sodium trifluoroacetate (*82*). Samples were injected at a rate of 5 μl/min. Two hundred scans were performed for each measurement. The root mean square value of the internal calibration was <0.1 parts per million.

Formulae were calculated in the *m*/*z* range of 200 to 650 considering the following elements: ^1^H, ^12^C, ^13^C_0–1_, ^16^O, ^14^N_0–4_, ^32^S_0–2_, ^34^S_0–1_, and ^31^P_0–2_. Only formulae in the range of O/C ratios of 0.01 to 1.2 and H/C ratios of 0.35 to 2.3 were considered in the calculation process, and unrealistic formulae in the DOM samples with N_4_P_2_, N_2_P_2_, S_2_P_2_, O_1_, and O_0_ were removed from the dataset. Multiple formulae were filtered as described by Koch (*83*). Less than five multiple assignments were presented in each sample. Peaks found in the list of contamination formulae (anthropogenic surfactants listed at www.terrabase-Inc.com) or in blank samples of solid-phase extraction [relative intensity (rInt) >0.1] were deleted.

During initial processing, peak magnitude was presented as rInt, which was calculated by normalization to the base peak. In the final dataset, the abundance of formulae is reported as rInt of an individual formula compared to the total intensity of all assigned formulae.

### Inorganic chemistry

The concentrations of ammonium and sulfate in the liquid phase were determined by photometry (QuickChem 8500, Lachat) and ion chromatography (882 Compact IC plus, Metrohm), respectively. Because no zinc was added to the samples to fix S^2−^, sulfide oxidation during storage cannot be excluded. Consequently, the determined sulfate concentration reflects the total sulfur content. Dissolved iron and manganese were analyzed by inductively coupled plasma optical emission spectrometry (Vista Pro CCD-simultaneous, Agilent).

### Calculations

For the series at 55° and 85°C, the biotic and abiotic production of acetate were calculated from the live, inhibited, and killed series. (i) Abiotic acetate production was derived from the killed series as *A*_abiotic production_ = *A*_15days_ − *A*_0day_, where *A* is the acetate concentration after 15 days of incubation and before incubation, respectively. (ii) The net production of acetate (*A*_net production_) was determined on the basis of the accumulation of acetate in the live series. (iii) The biotic consumption of acetate (*A*_biotic consumption_) derives from the difference between inhibited and alive series and represents a minimum value as only sulfate reducers were inhibited. (iv) Together, net production and biotic consumption of acetate provide information of the total acetate production *A*_total production_ = *A*_net production_ + *A*_biotic consumption_. (v) Last, total and abiotic acetate production yield information on the biotic production of acetate production *A*_biotic production_ = *A*_total production_ − *A*_abiotic production_.

### Composition of artificial seawater and supplements

Artificial seawater with 15 mM sulfate concentration was generated from 1 liter of Milli-Q water and the following salts: 0.682 g of KCl, 1.5 g of CaCl_2_·2H_2_O, 8.3 g of MgCl_2_·6H_2_O, 26.4 g of NaCl, 3.741 g of MgSO_4_·7H_2_O, and 0.099 g of KBr. To stimulate microbial activity, 1 liter of artificial seawater was complemented with 0.3 ml of trace element solution (0.5 mM H_3_BO_3_, 0.5 mM MnCl_2_·4H_2_O, 0.8 mM CoCl_2_·6H_2_O, 0.1 mM NiCl_2_·6H_2_O, 0.01 mM CuCl_2_·2H_2_O, 0.5 mM ZnSO_4_·7H_2_O, and 0.15 mM Na_2_MoO_4_·2H_2_O; acidified), 0.3 ml of thiamine solution (100 mg/liter in stock), 0.3 ml of vitamin B_12_ solution (50 mg/liter in stock), and 0.3 ml of vitamin mixture solution, according to a recipe adapted from Widdel and Bak (*84*). The vitamin mixture solution contained (10 mM sodium phosphate, 4 mg of amino benzoic acid, 1 mg of d-biotin, 10 mg of nicotinic acid, 5 mg of calcium d-pantothenate, 15 mg of pyridoxine dihydrochloride, 5 mg of riboflavin, 5 mg of thioctic acid, 4 mg of folic acid, and 1.5 mg of l-lipoic acid in 100 ml of stock solution). To buffer the pH value, 1 ml of NaHCO_3_ (aq, 1 M) was added to 1 liter of artificial seawater from a stock solution that had been sterilized by filtration with a 0.1-μm filter (polyethersulfone, Sartorius) and was kept under CO_2_.

### Use of artificial intelligence

During the preparation of this manuscript, we used artificial intelligence (AI)–powered language tools, specifically Perplexity, ChatGPT-4o, and DeepL, for assistance with text editing and language refinement. The AI was prompted with tasks such as “Check grammar and clarity, keep the change as minimal as possible,” “Make this sentence more concise and fluent without changing its meaning,” and “Rephrase this word to sound more prudent.” These tools were used to enhance clarity and readability, but all scientific content, analyses, and interpretations were generated and verified by the authors. No AI tools were used to produce scientific results or draw conclusions.
