# Supplementary material for: Moderate heating renders 7.8-million-year-old sedimentary organic matter bioavailable
Source: Sci Adv. 2025 Aug 20;11(34):eadw8638. doi: 10.1126/sciadv.adw8638 (PMC12366706; doi:10.1126/sciadv.adw8638)
Supplement: Supplementary file 1 — Figs. S1 to S10 Tables S1 and S2 [file sciadv.adw8638_sm.pdf]

Science Advances
AAAS

Supplementary Materials for

Moderate heating renders 7.8-million-year-old sedimentary organic matter bioavailable

Shuchai Gan *et al.*

Corresponding author: Shuchai Gan, ganshuchai@scbg.ac.cn

Sci. Adv. **11**, eadw8638 (2025)
DOI: 10.1126/sciadv.adw8638

This PDF file includes:

Figs. S1 to S10
Tables S1 and S2

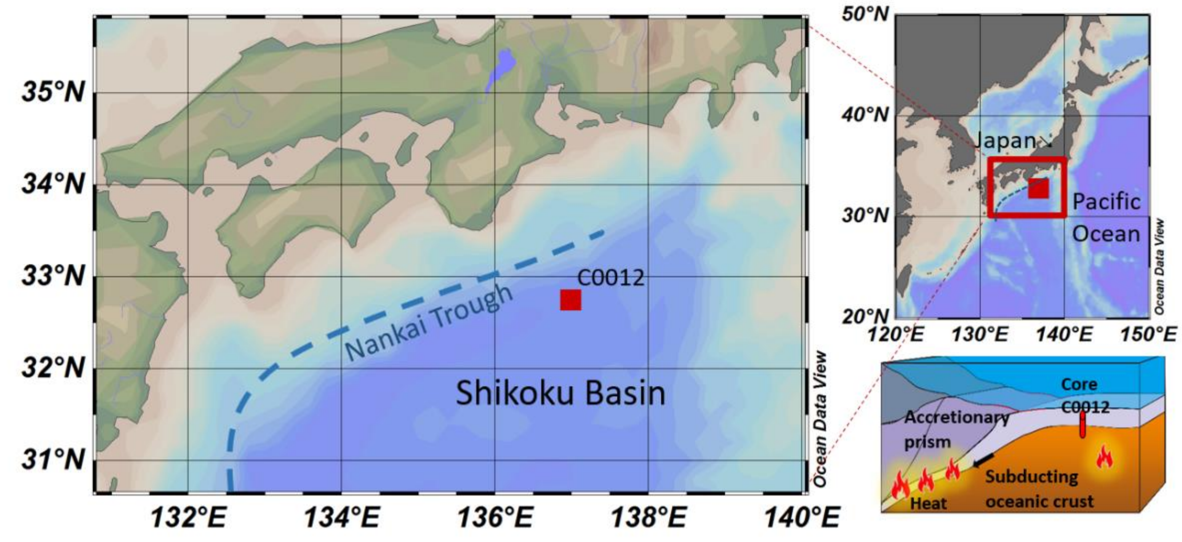

Fig. S1. Location of the sampling site and tectonic structure in the Nankai Trough.

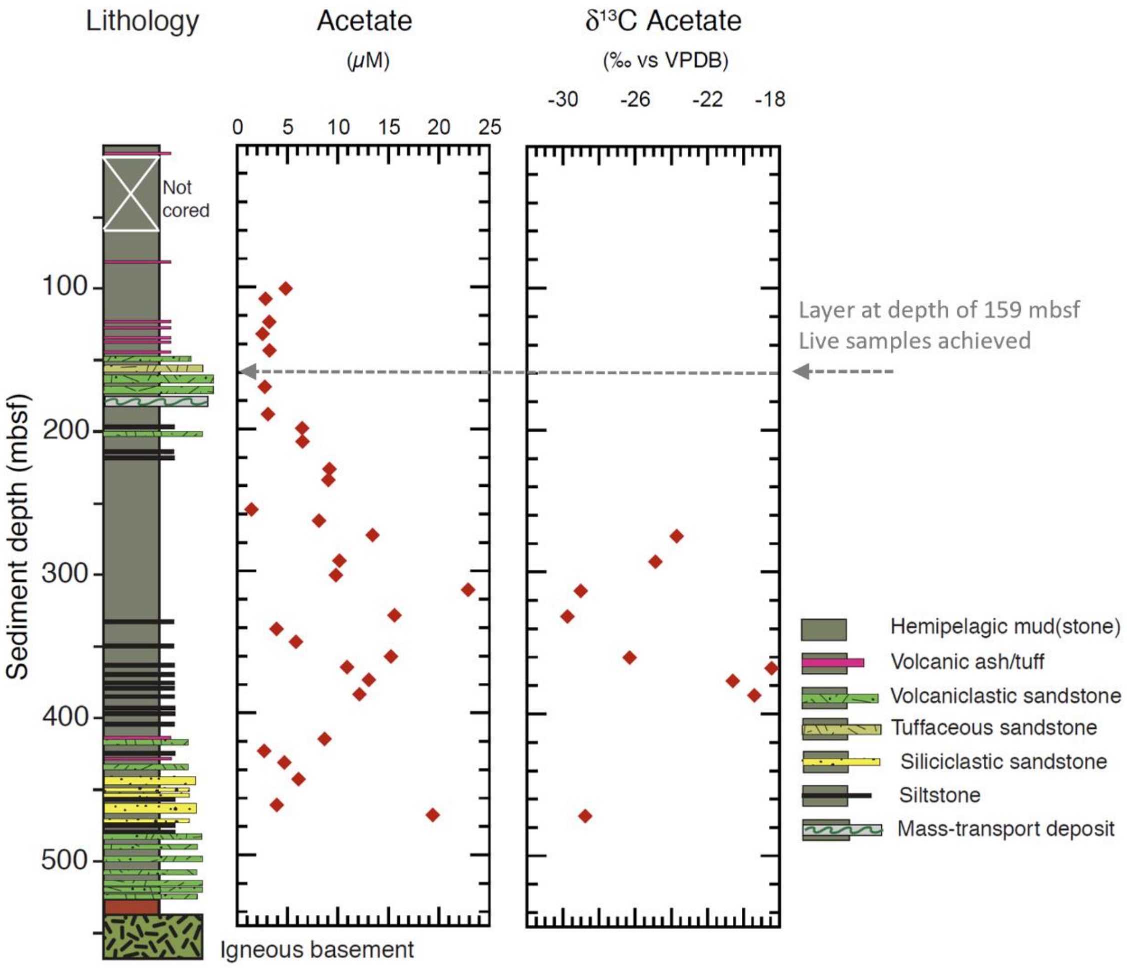

Fig. S2. *In situ* profile of acetate and lithology of the core. The arrow indicates the depth at which live samples were collected. Lithology was modified from Expedition 322 Scientists *(70)*.

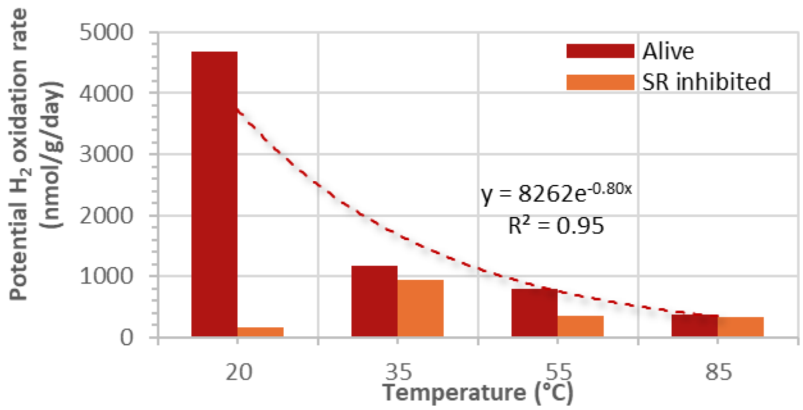

Fig. S3. Potential H_2 oxidation rate in alive and inhibited series on day 55. The red line represents the exponential trend with temperature for the alive series. The red and orange columns represent the live and SR-inhibited series, respectively.

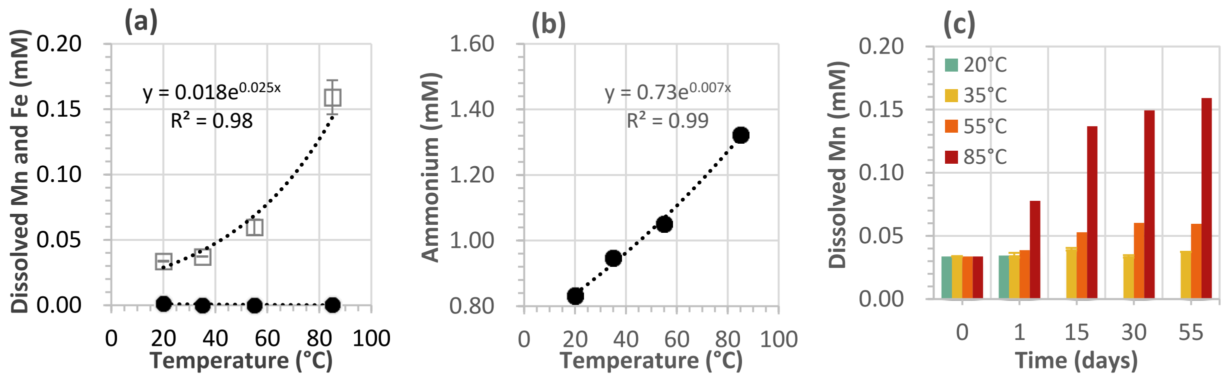

Fig. S4. Inorganics in the dissolved phase of all alive series on day 55: (a) dissolved manganese (squares) and iron (dots), (b) ammonia, and (c) increase of dissolved manganese in pore water during incubation. The dashed lines represent the exponential correlation with temperature.

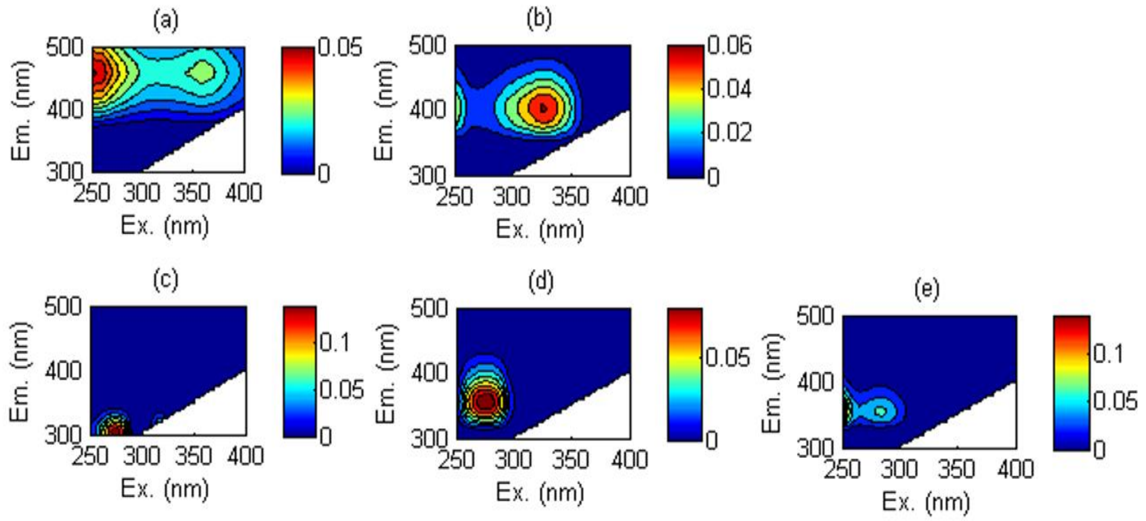

Fig. S5. **Five peaks were identified by PARAFAC analysis:** (a) humic-like peaks AC (ex/em: 325(250) nm/400 nm, (b) humic-like peak M (ex/em: 360(250) nm/460 nm), (c) protein-like peak (ex/em: 275 nm/310 nm, (d) protein-like peak (275 nm/350 nm), and (e) one peak consisting of a mixture of protein-like peaks and vitamins, as shown in the blank of artificial seawater with added vitamins (ex/em: 250(280) nm/350 nm). Total humic-like DOM, represented by peaks AC and M, are abbreviated as 'Hum'.

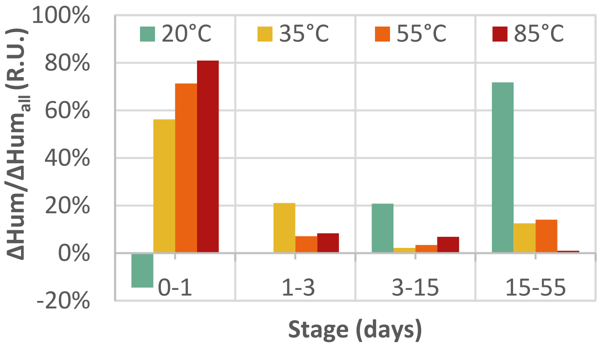

Fig. S6. Increase in humic-like DOM (ΔHum) in the live series normalized to that within 55 days. $\Delta Hum_{all} = Hum_{day55} - Hum_{day0}$.

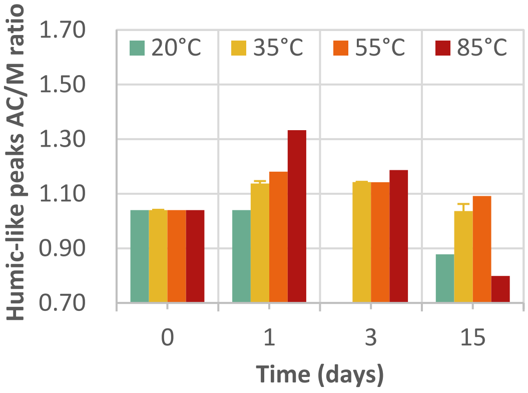

Fig. S7. **Variation in the ratio of humic-like peaks (AC/M) in the killed series.**

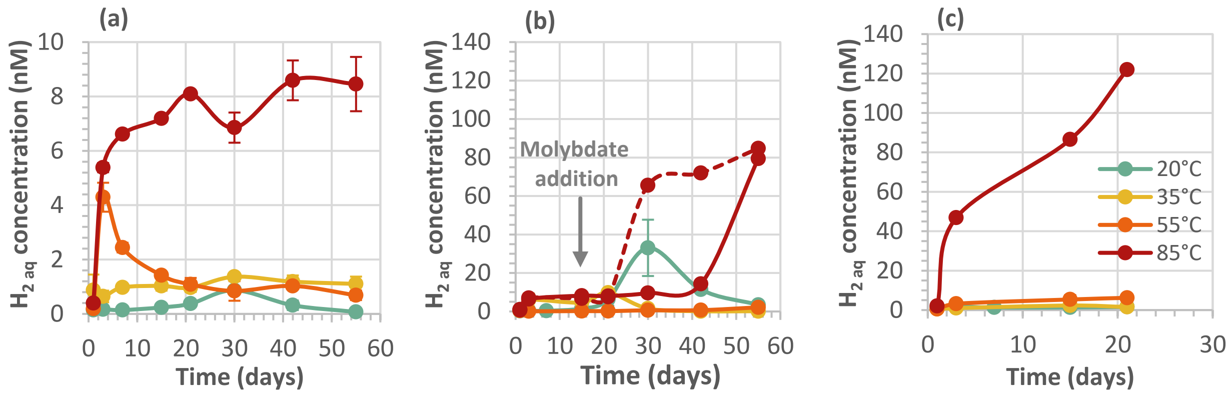

Fig. S8. **Concentration of H_2 during incubations**: (a) alive series; (b) inhibited series; (c) killed series. The dashed line in (b) shows replicates at 85°C. The arrow indicates the time point of molybdate addition to inhibit sulfate reducers. Note that the y-axis scale in (a) differs from the others.

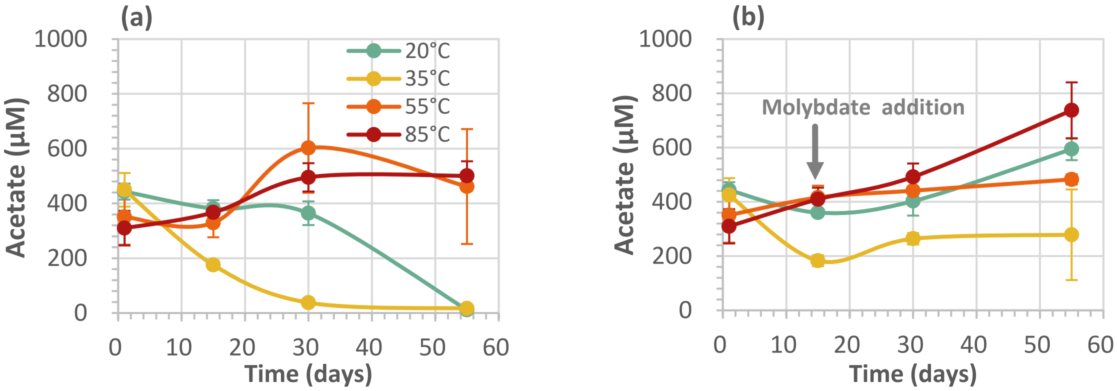

Fig. S9. **Acetate concentration in alive series during incubation**: (a) alive series (55 days); (b) inhibited series (55 days). The arrow indicates the time point of molybdate addition for inhibiting sulfate reducers.

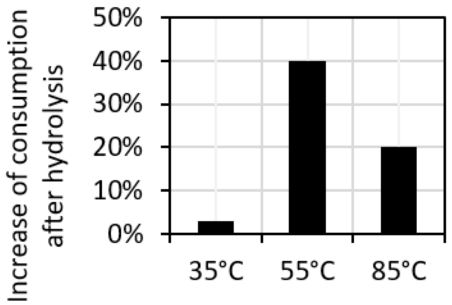

Fig. S10. Increased consumption of protein-like DOM in the YE (hydrolyzed) series. This series is used to test the rate of the intermediate step: monomer consumption. The most accelerated consumption by hydrolysis occurred at 55°C, suggesting that inhibition of hydrolysis is a limiting factor particularly at this temperature. YE = yeast extract.

Table S1. Incubation settings.

Series No.	Series name	Temperature of subseries	Duration (days) and duplicates
I	Control series	20°C	55, duplicates for all temperatures
II	Alive series	35°C, 55°C, 85°C	55, duplicates for all temperatures
III	Inhibited series	20°C, 35°C, 55°C, 85°C	55 (molybdate addition on 15th day), duplicates for all temperatures
IV	Killed series	20°C, 35°C, 55°C, 85°C	55 (data after 21 days were not interpreted), duplicates for all temperatures except 85°C
V	YE series	35°C, 55°C and 85°C	10, no duplicates
VI	Hydrolyzed-YE series	35°C, 55°C and 85°C	10, no duplicates

Table S2. Concentration corresponding to the normalized data presented in Figure 1. $Mn_{(aq)}$ on day 55 at 20 °C was estimated from 15-day average release data. AC and M represent humic-like DOM; P represents protein-like DOM.

Temperature	Day	$H_{2(aq)}$ (nM)	$Mn_{(aq)}$ (mM)	AC (R.U.)	M (R.U.)	P (R.U.)	Ammonium (mM)	Acetate (μM)
20°C	1	0.14	0.034	0.34	0.28	0.51	0.78	443
20°C	55	0.07	0.036	0.38	0.36	1.50	0.83	11
35°C	55	1.10	0.037	0.55	0.45	0.44	0.95	17
55°C	55	0.69	0.060	0.77	0.60	0.84	1.05	462
85°C	55	8.46	0.159	1.47	0.97	1.24	1.32	501
